# Efficacy of Ultrasound-Guided Injections in Patients Unable to Access or Benefit From Physical Therapy: A Comparative Study of Subacromial Corticosteroid Injection, Suprascapular Nerve Block, and Their Combination in Shoulder Impingement Syndrome

**DOI:** 10.7759/cureus.79008

**Published:** 2025-02-14

**Authors:** Alper Uysal

**Affiliations:** 1 Physical Medicine and Rehabilitation, Mersin City Training and Research Hospital, Mersin, TUR

**Keywords:** physical therapy barriers, rotator cuff tendinopathy, shoulder impingement syndrome, subacromial bursa steroid injection, subacromial impingement syndrome, suprascapular nerve block, ultrasound guided

## Abstract

Background

This study aims to compare the efficacy of ultrasound (US)-guided subacromial bursa steroid injection (SABSI), suprascapular nerve block (SSNB), and combined injection therapy (SABSI + SSNB) on pain and shoulder function in patients with subacromial impingement syndrome who either did not respond to physical medicine and rehabilitation (PM&R) or reported being unable to attend the PM&R unit regularly.

Methodology

In this prospective, comparative cohort study, patients were randomly assigned using the envelope method to ensure an unbiased distribution. In total, 95 patients with subacromial impingement syndrome were divided into three groups according to the injection technique applied. All injections were applied to Group 1 (SABSI), Group 2 (SSNB), and Group 3 (SABSI + SSNB) under US guidance. Pain and shoulder function levels of the groups before and after treatment at one month and three months were evaluated using the Visual Analog Scale (VAS) and the Disabilities of the Arm, Shoulder and Hand (DASH) scale, respectively. For statistical analysis, the Shapiro-Wilk test was used to assess the normality of continuous data. One-way analysis of variance with Bonferroni correction was applied for normally distributed variables, while the Kruskal-Wallis test was used for non-normally distributed data. Pearson’s chi-square test was performed to compare categorical variables.

Results

Between pre-treatment and one month post-treatment, there were statistically significant differences in the percentage change of VAS rest, VAS activity, and DASH scores among the groups (p = 0.004, <0.001, and <0.001, respectively). These significant differences persisted over the period from pre-treatment to three months post-treatment, with all p-values being ≤0.002. Throughout both assessment intervals, Groups 1 and 2 displayed statistically similar percentage changes across all parameters; however, these changes were significantly lower than those observed in Group 3. Patients unable to attend PM&R units displayed a longer symptom duration compared to patients who were non-responders to PM&R (p = 0.015). The distribution of these three injection techniques across the two groups was similar (p = 0.831), and both groups showed similar responses to US-guided injections in terms of pain and functionality (all p > 0.05).

Conclusions

US-guided SABSI combined with SSNB in patients with shoulder impingement was found to be superior compared to their sole applications in terms of improvement in shoulder pain and function. These findings suggest that the combined approach is a promising alternative for managing shoulder impingement syndrome, particularly in patients facing barriers to PM&R access.

## Introduction

Shoulder impingement syndrome is a condition characterized by shoulder pain and functional limitation resulting from the compression of the tendons of the rotator cuff, the long head of the biceps, and the subacromial bursa under the coracoacromial arch. Inflammation and degeneration of the tendons, inflammation of the bursa, rotator cuff weakness, posterior capsule tension of the shoulder, dysfunction of the scapula, and abnormalities of bone structure and soft tissue that narrow the subacromial space can cause shoulder impingement syndrome, alone or in combination [1].

Immobilization, cold, exercise, heat, electrotherapy, kinesio taping, manual therapy, acupuncture, elastic therapeutic tape, non-steroidal anti-inflammatory drugs (NSAIDs), cortisone injections, and surgery are used in the treatment of shoulder impingement syndrome [2]. Subacromial bursa steroid injections (SABSIs) are one of the most widely preferred treatments for rotator cuff syndrome, especially long-acting corticosteroids, such as triamcinolone acetonide. Corticosteroids have anti-inflammatory and pain-relieving properties [3].

Another effective option for the treatment of shoulder pain is suprascapular nerve block (SSNB). Roughly 70% of the sensory input transmission from the shoulder joint to the upper centers is attributed to the suprascapular nerve. SSNB reduces the sensation of pain through various mechanisms, such as the reduction of peripheral nociceptive inputs, peripheral and central sensitization, and substance P levels [4].

In a landmark (LM)-guided injection technique, it is difficult to ensure how deep the needle is. In addition, the accuracy of LM-guided injections is low, especially in obese patients who do not have a clear anatomical reference point. The LM-guided injection may result in an injury of the joint cartilage or rotator cuff tendon, and the associated rotator cuff rupture may develop in the long term. Ultrasound (US)-guided SABSI is becoming more common due to its advantages such as being inexpensive, fast, radiation-free, and the ability to visualize the needle during the injection procedure [5]. Furthermore, the role of US guidance in minimizing the risk of pneumothorax during SSNB is significant and should not be overlooked [6].

We hypothesize that a combination of SABSI and SSNB under ultrasound guidance will provide superior pain relief and functional improvement compared to either technique alone in the treatment of rotator cuff tendinopathy. To our knowledge, this is the first study to comprehensively compare these three modalities within a single framework. This study addresses an important clinical issue by being the first to investigate treatment options for patients with rotator cuff syndrome who face barriers to accessing physical medicine and rehabilitation (PM&R) services and fills a critical gap in the literature regarding alternative treatments for these patients.

## Materials and methods

Study design and participants

This prospective, randomized, comparative cohort study was conducted at the PM&R Clinic of Hatay Training and Research Hospital from November 2021 to October 2022. A total of 97 patients, aged between 20 and 70 years, who applied to the outpatient clinic and were diagnosed with shoulder impingement syndrome, were included in the study. The sample size determination was based on a previous study by Yılmaz [4]. This study used power analysis (power = 0.80, α = 0.05, β = 0.20) and found that 12 patients per group were sufficient to detect differences in Visual Analog Scale (VAS) scores [4]. Due to similarities in study design and scales, a comparable estimate was deemed appropriate, and the groups were set to include at least 30 patients each. The study was conducted in accordance with the principles outlined in the Declaration of Helsinki. Ethical approval for the research was granted by Hatay Mustafa Kemal University, with decision number 21 and protocol code 2021/54. Informed consent was obtained from all participants before their inclusion in the study.

Inclusion criteria

Patients with at least one positive physical examination test (painful arc, Hawkins, or Neer test) and evidence of inflammation in the rotator cuff tendons or subacromial bursa on magnetic resonance imaging were selected for the study. All patients included in the study were initially managed conservatively with muscle relaxants, NSAIDs, and a home exercise program. Patients who did not respond adequately to this initial conservative treatment, and additionally either did not respond to PM&R or stated that they could not attend the PM&R unit regularly, were included in the study.

Exclusion criteria

Rotator cuff tears (either partial or full thickness), severe glenohumeral joint osteoarthritis [7], adhesive capsulitis, a history of shoulder operation, a history of fracture in the upper extremity, presence of inflammatory rheumatologic disease, pregnancy, malignancy, infection, a history of shoulder injection, and use of anticoagulant drugs were the exclusion criteria for the study.

Randomization and formation of groups

**Figure 1:**
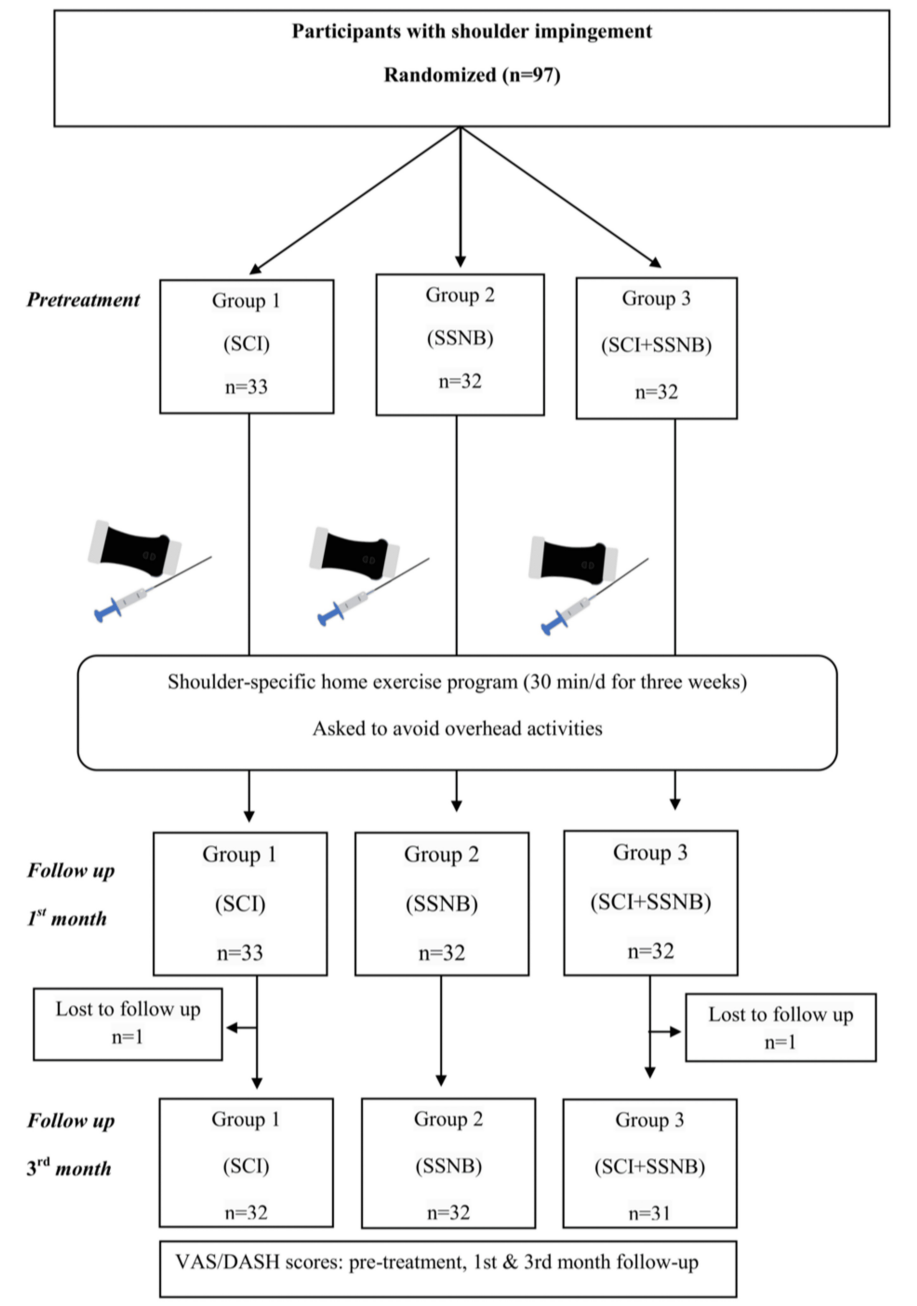
Flowchart of the study. SCI: subacromial corticosteroid injection; SSNB: suprascapular nerve block; VAS: Visual Analog Score; DASH: Disabilities of the Arm, Shoulder and Hand

The patients were randomly divided into three groups using a random envelope method to ensure an unbiased distribution: Group 1 (SABSI), Group 2 (SSNB), and Group 3 (SABSI + SSNB). Injections were administered to each group under US guidance by an experienced specialist (AU). All three groups were prescribed a shoulder-specific home exercise program, which included pendulum exercises, posterior capsule stretching exercises, passive and active-assisted range of motion exercises, and isometric strengthening exercises targeting the rotator cuff muscles, performed in three sets of 10 repetitions a day for three weeks (Figure 1).

Injection techniques and treatment protocols

The Clarius L7 HD wireless US device was used until May 2022. From this date onwards, the Clarius L7 HD3 wireless US device was used throughout the study.

SABSI Procedure

**Figure 2:**
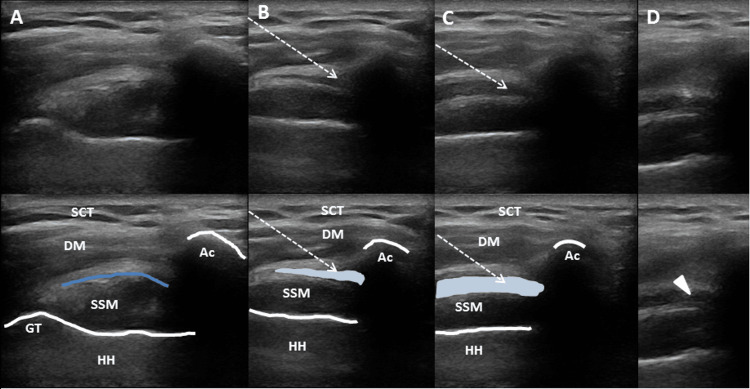
Step-by-step ultrasound images of the subacromial bursa filling with injection material during SABSI. (A) The area before SABSI. (B) The area during early SABSI. (C) The area during late SABSI. (D) The needle tip represented by a triangular arrowhead, indicating the final position within the bursa. The dashed arrow indicates the needle direction, the white lines represent the humeral head cortex, the dark blue line shows the subacromial bursa, and the light blue area illustrates the spread of the injection material within the bursa. SABSI: subacromial bursa steroid injection; SCT: subcutaneous tissue; DM: deltoid muscle; Ac: acromion bone; SSM: supraspinatus muscle; GT: greater trochanter; HH: humeral head

SABSI was performed with the patient in a sitting position using a lateral approach under US guidance. After detecting the subacromial bursa on the lateral side of the shoulder, it was reached by passing the skin, subcutaneous fat tissue, and deltoid muscle under sterile conditions by monitoring the needle tip with US [8]. Subsequently, 1 mL of 20 mg triamcinolone hexacetonide, 1 mL of 2% lidocaine, and 3 mL of saline (SF) mixture were injected with a 22-gauge needle tip (Figure 2). A systematic review and meta-analysis published in 2017 assessed the use of corticosteroid and anesthetic agents in subacromial injections for treating impingement syndrome. The majority of the studies included in the review utilized triamcinolone combined with lidocaine for these injections. The analysis compared injection volumes, using 5 mL as the threshold, and found no significant difference in therapeutic efficacy between high-volume (≥5 mL) and low-volume (<5 mL) injections and indicated that there was no significant difference in the efficacy between the two different doses (20 and 40 mg) of corticosteroid injections. Based on these results, a total injection volume of 5 mL was preferred, with triamcinolone (20 mg) and lidocaine being the agents of choice for the study [9]. Thus, the present study was planned based on this information.

SSNB Procedure

**Figure 3:**
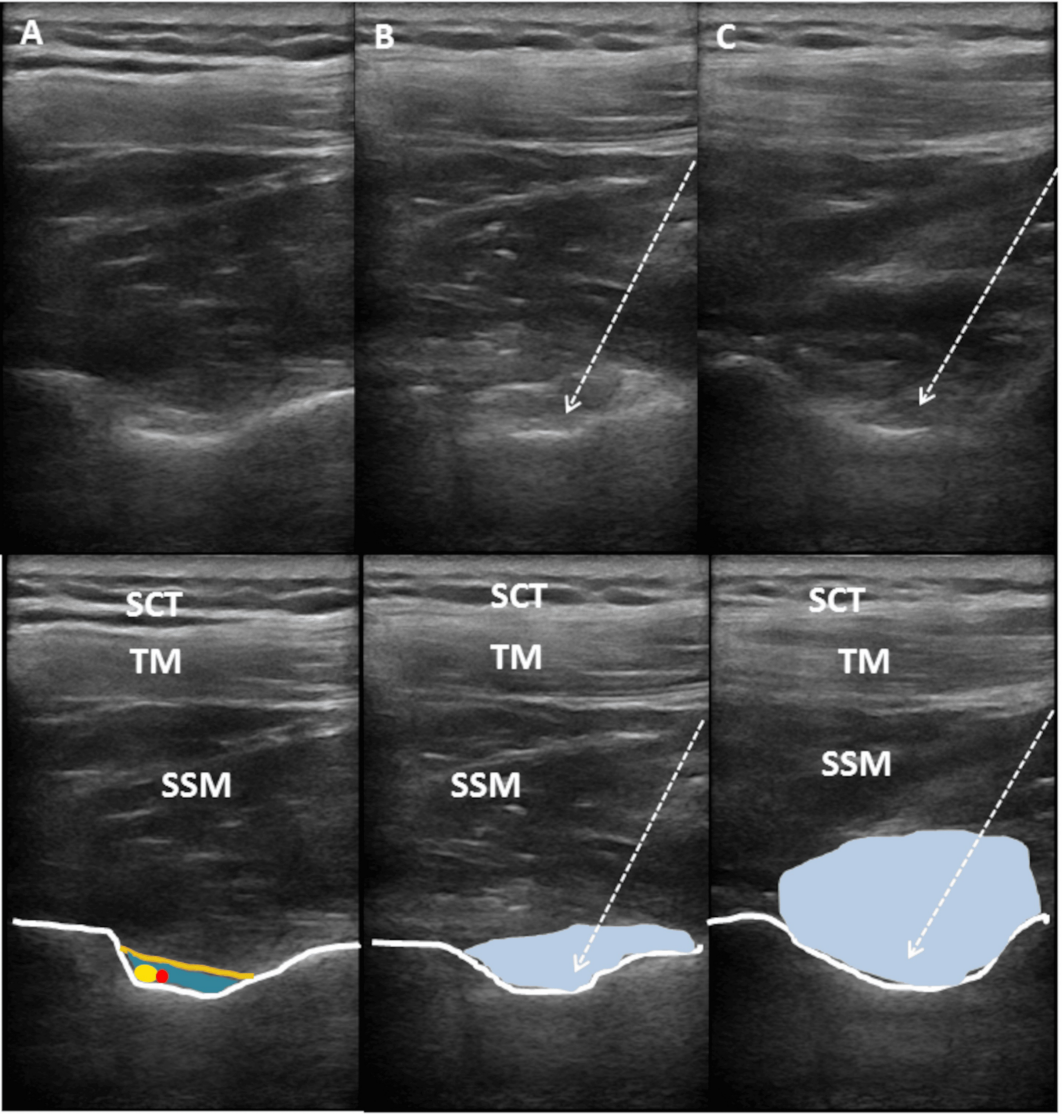
Step-by-step ultrasound images of the suprascapular notch filling with injection material during SSNB. (A) The area before injection. (B) The are during early injection. (C) The are during late injection. The dashed arrow represents the needle and its direction. The white lines at the bottom represent the cortex of the scapular bone, and the brown line represents the transverse scapular ligament. The dark blue area indicates the suprascapular notch, the yellow ellipse represents the suprascapular nerve, the red circle represents the suprascapular artery, and the light blue area shows the spread of the injection material within the notch and then into the fossa. SSNB: suprascapular nerve block; SCT: subcutaneous tissue; TM: trapezius muscle; SSM: supraspinatus muscle

SSNB was performed using a posterior approach with the patient in a seated position [10]. After the suprascapular notch and overlying transverse scapular ligament were found, under sterile conditions, the skin, subcutaneous tissue, trapezius and supraspinatus muscles, and, finally, the transverse scapular ligament were passed using a 22-gauge needle under US guidance with an in-plain technique. During the procedure, care was taken to avoid injuring the suprascapular artery and nerve, which lie beneath the transverse scapular ligament within the suprascapular notch. Then, a mixture of 1 mL (20 mg) triamcinolone hexacetonide and 5 mL 2% lidocaine was injected into the suprascapular notch, beneath the transverse scapular ligament, and next to the suprascapular nerve. Thus, the suprascapular nerve located there was blocked [10] (Figure 3). Although the literature is inconclusive, SSNB for chronic shoulder pain is often performed using a combination of local anesthetics and steroids to prolong the block’s effects. In this study, lidocaine, a commonly used local anesthetic, was administered in combination with triamcinolone. The doses employed in this research were consistent with the dosage ranges reported in the existing literature [11]. According to studies in the literature, the volume of injectate for SSNB typically ranges between 2 and 10 mL [11]. In one study, different volumes were used for each shoulder: 10 mL for the right shoulder and 5 mL for the left. The findings suggested that 5 mL was sufficient to fill the lateral half of the supraspinatus fossa and effectively block the suprascapular nerve located in that area [12]. Therefore, the present study was designed based on these findings.

Outcome measures and evaluation times

The gender, age, body mass index (BMI), symptom duration, dominant arm, affected arm, and the reasons for inclusion such as non-responders to PM&R or an inability to attend the PM&R unit were recorded for all individuals participating in this study. Pain intensity was evaluated using VAS, and shoulder disability level was assessed using the Disabilities of the Arm, Shoulder and Hand (DASH) scale. The validity and reliability of these measures have been established by Boonstra et al. [13] and Beaton et al. [14], respectively. All three groups were evaluated in terms of the above parameters (VAS rest, VAS activity, and DASH) before the treatment, the first month after the treatment, and the third month after the treatment (Figure 1). Thus, the effects of these treatments on the level of pain and shoulder disability were examined.

Statistical analysis

To assess whether continuous data conformed to the assumption of normality, the Shapiro-Wilk test and the coefficient of variation were applied. Continuous data with normal distribution were presented as arithmetic mean ± standard deviation, and continuous data without normal distribution were presented as median (minimum-maximum). Categorical variables were expressed as both frequency counts and percentages. The Pearson’s chi-square Test was conducted to identify statistical differences between groups with respect to categorical variables. Group differences in continuous variables were analyzed using a one-way analysis of variance and Bonferroni correction if the assumption of normality was met, and the Kruskal Wallis Test if not. For analyses, a significance threshold of p < 0.05 was set. SPSS version 22 (IBM Corp., Armonk, NY, USA) was used for data analysis.

## Results

No statistically significant differences were observed between the groups with respect to gender, age, BMI, dominant arm, affected arm, and reasons for inclusion of patients at baseline (p > 0.05) (Table 1).

**Table 1:** Comparison of demographic and clinical characteristics of the injection groups at baseline. *: Pearson’s chi-square test; **: one-way analysis of variance. n: number; SD: standard deviation; BMI: body mass index; PM&R: physical medicine and rehabilitation

		Group 1 (n = 32)	Group 2 (n = 32)	Group 3 (n = 31)	P-value
Gender	Female, n (%)	24 (75.0)	23 (71.9)	24 (77.4)	0.879*
	Male, n (%)	8 (25.0)	9 (28.1)	7 (22.6)	
Age (mean ± SD)		52.84 ± 11.28	54.66 ± 9.78	55.65 ± 6.81	0.496**
BMI (kg/m²), mean ± SD		28.11 ± 3.43	28.16 ± 2.55	28.90 ± 2.14	0.457**
Symptom duration (months), mean ± SD		8.03 ± 3.56	8.74 ± 3.26	7.97 ± 2.89	0.581**
Dominant arm	Right, n (%)	28 (87.5)	27 (84.4)	28 (90.3)	0.777*
	Left, n (%)	4 (12.5)	5 (15.6)	3 (9.7)	
Affected arm	Right, n (%)	20 (62.5)	20 (62.5)	22 (71.0)	0.719*
	Left, n (%)	12 (37.5)	12 (37.5)	9 (29.0)	
Reasons for inclusion of patients	Non-responders to PM&R, n (%)	10 (31.25)	8 (25)	8 (25.8)	0.831*
	Patients unable to attend PM&R unit, n (%)	22 (68.75)	24 (75)	23 (74.2)	

The baseline VAS rest, VAS activity, and DASH scores were statistically similar across the three groups (p > 0.05). At the one-month follow-up, VAS rest scores remained similar between the groups (p = 0.056); however, by the third-month follow-up, VAS rest scores (p = 0.019), as well as VAS activity and DASH scores at both one and three months (p < 0.001), demonstrated statistically significant differences between the groups. In these parameters, Group 3 exhibited lower values compared to Groups 1 and 2, with no statistical difference observed between Groups 1 and 2 (Table 2).

**Table 2:** Comparison of VAS and DASH scores of the injection groups. *: Kruskal-Wallis test; **: one-way analysis of variance; ***: the difference between the means with different lowercase letters in the same line was found to be statistically significant (p < 0.05). n: number; SD: standard deviation; min: minimum; max: maximum; VAS: Visual Analog Scale; DASH: Disabilities of the Arm, Shoulder and Hand

	Group 1 (n = 32)	Group 2 (n = 32)	Group 3 (n = 31)	P-value
VAS rest (baseline), mean ± SD	3.69 ± 1.71	3.78 ± 1.88	3.80 ± 1.72	0.961**
VAS rest (1st month), median (min-max)	1.0 (0.0-4.0)	1.0 (0.0-5.0)	0.0 (0.0-3.0)	0.056*
VAS rest (3rd month), median (min-max)	1.0 (0.0-5.0)^a^	1.0 (0.0-4.0)^a^	0.0 (0.0-3.0)^b^	0.019*
VAS activity (baseline), median (min-max)	7.50 (5.0-10.0)	8.0 (5.0-10.0)	8.0 (5.0-10.0)	0.787*
VAS activity (1st month), median (min-max)	4.0 (0.0-8.0)^a^	4.0 (0.0-7.0)^a^	2.0 (0.0-4.0)^b^	<0.001*
VAS activity (3rd month), median (min-max)	4.0 (0.0-8.0)^a^	4.0 (1.0-7.0)^a^	2.0 (0.0-5.0)^b^	<0.001*
DASH (baseline), mean ± SD	57.53 ± 13.46	58.56 ± 13.22	61.41 ± 15.04	0.521**
DASH (1st month), mean ± SD	31.72 ± 11.99^a^	34.62 ± 10.95^a^	17.83 ± 10.73^b^	<0.001**
DASH (3rd month), median (min-max)	29.17 (4.17-53.33)^a^	33.33 (2.50-56.67)^a^	19.67 (0.0-48.83)^b^	<0.001*

The percentage change in VAS rest, VAS activity, and DASH scores between pre-treatment and one month post-treatment showed statistically significant differences between the groups (p = 0.004, <0.001, <0.001, respectively). Similarly, the percentage changes between pre-treatment and three months post-treatment also demonstrated significant differences across the groups (all p ≤ 0.002). At both time intervals, the percentage changes for all parameters in Groups 1 and 2 were statistically similar, while these changes were significantly lower than those observed in Group 3 (Table 3).

**Table 3:** Comparison of percentage change in VAS and DASH scores among the injection groups. *: Kruskal-Wallis Test; **: one-way analysis of variance; ***: a statistically significant difference was identified between values labeled with different lowercase letters on the same line (p < 0.05). n: number; SD: standard deviation; min: minimum; max: maximum; BT: before treatmant; AT: after treatment; VAS: Visual Analog Scale; DASH: Disabilities of the Arm, Shoulder and Hand

	Group 1 (n = 32)	Group 2 (n = 32)	Group 3 (n = 31)	P-value
VAS rest (BT–1 month AT), median (min/max)	-66.6 (-100.0/0.0)^a^	-66.6 (-100.0/25.0)^a^	-80.0 (-100.0/0.0)^b^	0.004*
VAS rest (BT–3 months AT), median (min/max)	-66.6 (-100.0/0.0)^a^	-70.83 (-100.0/0.0)^a^	-100.0 (-100.0/0.0)^b^	0.002*
VAS activity (BT–1 month AT), mean ± SD	-50.58 ± 17.51^a ^	-47.28 ± 18.15^a^	-75.46 ± 13.53^b^	<0.001**
VAS activity (BT–3 months AT), mean ± SD	-49.70 ± 20.14^a^	-50.34 ± 16.29^a^	-74.50 ± 14.44^b^	<0.001**
DASH (BT–1 month AT), mean ± SD	-45.37 ± 15.36^a^	-40.88 ± 15.36^a^	-72.96 ± 12.75^b^	<0.001**
DASH (BT–3 months AT), mean ± SD	-46.04 (-91.11/-8.62)^a^	-38.01 (-95.45/-17.07)^a^	-67.19 (-100/-42.62)^b^	<0.001*

No statistically significant differences were observed between the groups with respect to gender, age, BMI, dominant arm, affected arm, and injection technique applied (p > 0.05), except for symptom duration (p = 0.015) (Table 4).

**Table 4:** Comparison of demographic and clinical characteristics of the groups at baseline. *: Pearson’s chi-square test; **: independent-samples t-test. n: number; SD: standard deviation; BMI: body mass index; PM&R: physical medicine and rehabilitation; SABSI: subacromial bursa steroid injection; SSNB: suprascapular nerve block

		Non-responders to PM&R (n = 26)	Patients unable to attend the PM&R unit (n = 69)	P-value
Gender	Female, n (%)	17 (34.6)	54 (78.3)	0.198*
	Male, n (%)	9 (65.4)	15 (21.7)	
Age, mean ± SD		51.12 ± 10.87	55.59 ± 8.66	0.067**
BMI (kg/m²), mean ± SD		28.18 ± 3.34	28.46 ± 2.54	0.700**
Symptom duration (months), mean ± SD		6.84 ± 3.40	8.77 ± 3.03	0.015**
Dominant arm	Right, n (%)	23 (88.5)	60 (87)	0.844*
	Left, n (%)	3 (11.5)	9 (13)	
Affected arm	Right, n (%)	19 (73.1)	43 (62.3)	0.326*
	Left, n (%)	7 (26.9)	26 (37.7)	
Injection technique applied	SABSI, n (%)	10 (38.5)	22 (31.9)	0.831*
	SSNB, n (%)	8 (30.8)	24 (34.8)	
	SABSI + SSNB, n (%)	8 (30.8)	23 (33.3)	

VAS rest, VAS activity, and DASH scores at baseline, first month, and third month were similar between the groups (p > 0.05) (Table 5).

**Table 5:** Comparison of VAS and DASH scores of the groups. *: Mann-Whitney U Test; **: independent-samples t-test. n: number; SD: standard deviation; min: minimum; max: maximum; VAS: Visual Analog Scale; DASH: Disabilities of the Arm, Shoulder and Hand; PM&R: physical medicine and rehabilitation

	Non-responders to PM&R (n = 26)	Patients unable to attend the PM&R unit (n = 69)	P-value
VAS rest (baseline), median (min-max)	3.5 (1.0-7.0)	4.0 (0.0-8.0)	0.629*
VAS rest (1st month), median (min-max)	1.0 (0.0-3.0)	1.0 (0.0-5.0)	0.881*
VAS rest (3rd month), median (min-max)	1.0 (0.0-4.0)	1.0 (0.0-5.0)	0.692*
VAS activity (baseline), median (min-max)	8.0 (5.0-9.0)	8.0 (5.0-10.0)	0.569*
VAS activity (1st month), median (min-max)	4.0 (0.0-6.0)	3.0 (0.0-8.0)	0.623*
VAS activity (3rd month), median (min-max)	4.0 (0.0-8.0)	3.0 (0.0-7.0)	0.213*
DASH (baseline), mean ± SD	60.27 ± 14.82	58.72 ± 13.59	0.646**
DASH (1st month), mean ± SD	29.81 ± 12.37	27.55 ± 13.70	0.445**
DASH (3rd month), mean ± SD	31.77 ± 13.56	26.02 ± 12.87	0.069**

The percentage change in VAS rest, VAS activity, and DASH scores between pre-treatment and one month post-treatment or pre-treatment and three months post-treatment showed no statistically significant difference between the groups (p > 0.05) (Table 6).

**Table 6:** Comparison of percentage change in VAS and DASH scores among the groups. *: Mann-Whitney U test; **: independent-samples t-test. n: number;  SD: standard deviation; min: minimum; max: maximum; BT: before treatmant; AT: after treatment; VAS: Visual Analog Scale; DASH: Disabilities of the Arm, Shoulder and Hand; PM&R: physical medicine and rehabilitation

	Non-responders to PM&R (n = 26)	Patients unable to attend the PM&R unit (n = 69)	P-value
VAS rest (BT–1 month AT), median (min/max)	-70.83 (-100.0/-40.0)	-75.00 (-100.0/25.0)	0.746*
VAS rest (BT–3 months AT), median (min/max)	-77.5 (-100.0/-20.0)	-75.00 (-100.0/0.0)	0.444*
VAS activity (BT–1 month AT), mean ± SD	-57.26 ± 17.58	-57.71 ± 21.82	0.917**
VAS activity (BT–3 months AT), mean ± SD	-53.47 ± 21.63	-59.72 ± 19.98	0.208**
DASH (BT–1 month AT), mean ± SD	-50.89 ± 18.60	-53.61 ± 20.88	0.541**
DASH (BT–3 months AT), median (min/max)	-45.05 (-100.0/-8.62)	-51.67 (-100.0/-17.07)	0.053*

## Discussion

While VAS rest, VAS activity, and DASH scores were comparable across groups at the start, significant improvements were observed by the third-month follow-up, especially in Group 3. Group 3 exhibited significantly greater improvements in pain and function compared to Groups 1 and 2, with the latter two showing no significant differences between each other. These findings highlight the superior effectiveness of the US-guided combined intervention applied in Group 3 for reducing pain and improving shoulder function over time.

Additionally, subgroup analysis was performed to compare patients who were non-responders to PM&R with those unable to attend PM&R units regularly. Patients unable to attend PM&R units displayed a longer symptom duration, indicating potential delays in accessing care. This situation could stem from barriers to the PM&R unit, such as logistical challenges (e.g., distance) and socioeconomic factors (e.g., difficulty in taking leave from work).

The two subgroups did not exhibit statistically significant differences in VAS rest, VAS activity, or DASH scores at baseline, first-month, or third-month follow-up. Similarly, the percentage change in these parameters between pre-treatment and post-treatment evaluations showed no significant differences. These findings suggested that the observed improvements in pain and function from the interventions are independent of the reasons for PM&R inaccessibility or non-responding. The present study underscored the need for healthcare policies that promote the integration of US-guided interventions, particularly for patients with limited access to PM&R due to socioeconomic or geographic barriers. The precision and safety of US guidance enhance the efficacy of these minimally invasive techniques, making them a valuable option for patients unresponsive to conservative treatments.

Chronic shoulder pain, the third most prevalent musculoskeletal condition, is a common disorder that not only leads to pain and functional limitations but also brings about socioeconomic consequences due to workforce loss [2]. The most commonly diagnosed condition in patients with chronic shoulder pain is subacromial syndrome, with rotator cuff tendinitis being particularly notable [15]. Therefore, effective treatment of this condition is crucial.

Especially in cases where patients do not respond adequately to initial conservative treatments, including exercise and pharmacological interventions, further treatment with PM&R agents in a hospital setting is usually recommended, as physical rehabilitation is often the first-line treatment for shoulder pain [11]. However, some patients can report that they cannot come to regular sessions due to work commitments or distance, while others may feel that they do not benefit sufficiently from the PM&R interventions prescribed. In such cases, minimally invasive methods such as injections may be preferred over more invasive treatment options such as surgery, as non-operative treatment is a first-line option [16]. The present study is significant in comparing the effectiveness of commonly used injection methods, such as SABSI and SSNB, as well as the combined application of both, in cases like these. While a single visit to a healthcare facility is sufficient for an injection, PM&R requires patients to visit the facility consecutively for 10-15 days. This can pose significant logistical challenges, including transportation costs, time investment, and potential loss of workdays. Some patients can report that they cannot come to PM&R sessions due to these barriers. The current study is valuable in highlighting this issue and providing a potential solution.

Corticosteroids, such as triamcinolone, exert both anti-inflammatory and direct analgesic effects by reducing pro-inflammatory mediators and influencing the cells involved in inflammatory responses when used for subacromial injection. In contrast, local anesthetics such as lidocaine act through membrane stabilization, selectively blocking small fibers responsible for transmitting pain and autonomic signals. Despite their differing pharmacological mechanisms, both corticosteroids and local anesthetics produce comparable outcomes in terms of pain relief and subsequent improvements in strength and upper limb function. In clinical practice, however, physicians commonly utilize a combination of corticosteroid suspensions and local anesthetics during local soft tissue injections to maximize therapeutic efficacy [9].

SSNB injections present a potential alternative to subacromial steroid injections for some patients. The suprascapular nerve is a mixed nerve, comprising both motor and sensory fibers, and is responsible for transmitting around 70% of the sensory input to the shoulder region. Injections targeting the suprascapular nerve are employed in the treatment of various chronic shoulder disorders. However, the precise mechanisms underlying the clinical efficacy of SSNB injections remain unclear [11].

SSNB can play a crucial role in diminishing neurogenic inflammation linked to local inflammatory processes, particularly those regulated through neuromodulators, such as calcitonin gene-related peptide and substance P. In addition, SSNB interrupts the afferent component of the abnormal reflex arcs involved in the development of certain chronic pain syndromes. This technique is also beneficial in breaking the persistent pain cycle commonly observed in such conditions. Moreover, SSNB has an impact on pain sensitization, which further enhances its clinical effectiveness [4].

Combination therapy can be highly valuable in managing such conditions, as it may allow for synergistic therapeutic effects through different mechanisms of action.

Coory et al. compared the clinical efficacy of US-guided SSNB and US-guided SABSI in patients with symptomatic rotator cuff tears and observed that SSNB was more effective than SABSI on pain and functional status [17]. Konar et al. compared the efficacy of LM-guided SABSI (methylprednisolone acetate and lignocaine mixture) and LM-guided SSNB (methylprednisolone acetate, lignocaine, and bupivacaine mixture) in patients with shoulder impingement syndrome. They observed that SSNB was more effective in improving pain and shoulder function compared to SABSI [18].

Sağlam et al. investigated the efficacy of SABSI (lidocaine and methylprednisolone acetate mixture) and SSNB (lidocaine and methylprednisolone acetate mixture) in patients with chronic shoulder pain. In contrast to Konar et al. and Coory et al., they found that the efficacy of the two injection techniques was similar [19].

Taskaynatan et al. evaluated the efficacy of LM-guided SABSI (lidocaine and methylprednisolone acetate mixture) and LM-guided SSNB ( lidocaine) on pain, disability, and range of motion in patients with shoulder pain. They found both treatment methods to be similar [20]. Although the literature still lacks consensus on whether SABSI or SSNB is superior, this study found that US-guided SABSI and US-guided SSNB were not superior to each other in terms of pain and function.

Yılmaz compared the efficacy of LM-guided SABSI alone with the combination of LM-guided SABSI and SSNB in patients with shoulder impingement syndrome and found that combined injection therapy provided better improvement in pain and shoulder function [4]. In support of Yılmaz’s findings, this study demonstrated that US-guided combined injection therapy provided better results in terms of shoulder pain and function compared to US-guided SABSI or SSNB alone.

Advancements in technology have positioned dynamic musculoskeletal US as an invaluable supplementary tool across various clinical specialties, including orthopedic surgery, PM&R, and sports medicine. Its capacity to provide high-resolution, real-time imaging has broadened its application, offering significant support in both the evaluation of soft tissue injuries and the treatment of these disorders by guiding procedures [21].

The use of US-guided injections is particularly beneficial, as it ensures visualization of the needle and the injectate in the targeted site, minimizing the risk of injury to nerves, blood vessels, and soft tissues such as tendons and muscles. Although the occurrence of pneumothorax during the SSNB procedure is documented to be less than 1%, it represents a noteworthy source of litigation in procedures performed for chronic pain, making up 5% of all legal claims [5,6,22]. A vertical approach to the suprascapular notch may increase the risk of pneumothorax. Possible complications of SSNB can be effectively reduced by using US guidance [6].

LM-guided injection technique may be less successful in obese patients. It is more difficult to detect bone landmarks and reach the target during injection because the subcutaneous adipose tissue is thick [5]. The palpation of the body reference points is also difficult for the application of the LM-guided injection technique in patients who have excess muscle tissue [23]. Although there is still no consensus in the literature on the superiority of US-guided injections over LM-guided injections for SABSI or SSNB [6,10,22-28], the US-guided injection method was preferred in this study due to its potential advantages.

A meta-analysis published in 2022 reported that US-guided SABSI was not superior to the LM-guided technique for improvement in pain and function [24]. Akbari et al. found that LM-guided and US-guided SABSIs were similarly effective in patients with subacromial impingement syndrome in terms of pain, range of motion, and function improvements [25]. Ayekoloye et al. also found that LM-guided and US-guided SABSIs in patients with subacromial impingement had similar efficacy in terms of pain and functional improvements [26].

In contrast, Azadvari et al. also compared the efficacy of LM-guided and US-guided SABSIs in patients with spinal cord sequelae with subacromial impingement. Although they found improvement in pain and function parameters in both groups, they observed that US-guided injections were superior [27]. According to a meta-analysis published in 2015, US-guided SABSIs in adults with shoulder pain are significantly more effective than LM-guided SABSIs [5].

Kamal et al. compared the efficacy of LM-guided and US-guided SSNB and found that the two techniques provided equal improvement in pain, range of motion, and shoulder function [28]. Sağlam and Alisar evaluated the efficacy of LM-guided and US-guided SSNB in chronic shoulder pain and found no difference between the two groups [29].

In contrast to Kamal et al. and Sağlam and Alisar, Gorthi et al. evaluated the efficacy of LM-guided and US-guided SSNB in patients with shoulder pain and observed that US-guided SSNB was more effective for improvement in pain and shoulder functions. In addition, they observed vascular injury in two patients and nerve injury in three patients in the LM-guided SSNB group. However, they did not encounter any complications in the US-guided SSNB group [10]. A 2023 meta-analysis compared the effectiveness of US-guided and LM-guided corticosteroid injections for shoulder pain. The results showed that US-guided injections provided greater pain relief, improved shoulder function, and increased abduction at six weeks. Despite similar outcomes on shoulder disability scales, the study recommended US guidance due to its advantages in needle visualization and reduced complication risk [30].

Previous studies have not specifically addressed the concepts of “non-responders to PM&R” and “patients unable to attend PM&R units” when evaluating patients. The subgroup of “patients unable to attend PM&R units” has been largely overlooked in the existing literature [16-18,23,25-27]. This study addresses this gap by identifying that patients who cannot attend PM&R have longer symptom duration, which may lead to prolonged work loss and greater impairment in daily activities. Additionally, the similar VAS and DASH score changes between subgroups support the generalizability of these minimally invasive treatments across diverse patient populations, regardless of their access to PM&R. Another major strength of the study is its prospective design and the fact that it is the first study to compare three different injection techniques (SABSI, SSNB, and SABSI + SSNB) in a single clinical framework under US guidance. US guidance not only improves needle placement accuracy but also reduces the risk of complications. These advantages make this approach clinically safer and relevant. Despite these strengths, the study has some limitations. The relatively small number of patients was the first limitation of the study. The second limitation of the study was that there was no exercise group as the control group. The short follow-up period after treatment was another limitation of the study. The final limitation of the study was the lack of a group benefiting from traditional PM&R. Future research should focus on long-term follow-up to evaluate the sustained benefits of these injections. Additionally, larger, multicenter randomized controlled trials should be conducted to further confirm the superiority of combined injection therapy. Further studies should also investigate the integration of US-guided interventions into routine clinical practice and assess their cost-effectiveness compared to traditional rehabilitation approaches.

## Conclusions

US-guided SSNB and SABSI, when applied together, provided superior pain relief and functional improvement in subacromial impingement syndrome compared to their applications alone. Additionally, subgroup analysis showed that these interventions were equally effective in patients unable to attend PM&R units and those non-responsive to PM&R. These findings highlight the potential of US-guided injections as a viable treatment alternative.
